# Efficacy and safety of remimazolam versus propofol in elderly patients undergoing gastrointestinal endoscopy: a systematic review and meta-analysis

**DOI:** 10.1097/MS9.0000000000005092

**Published:** 2026-06-01

**Authors:** Saba Aliha, Asad Bilal, Gauhar Hamid, Amira Shahid Sheikh, Salman Shafiq, Muhammad Shazad, Muhammad Tayyab, Ahmad Kamal, Usama Idrees, Muhammad Talha Naeem, Mohammad Ebad Ur Rehman, Huzaifa Ahmad Cheema, Asma’a Munasar Ali Alsubari, Asim Ahmad Khan

**Affiliations:** a Department of Medicine, Multan Medical and Dental College, Multan, Pakistan; b Department of Medicine, Nishtar Medical University, Multan, Pakistan; c Department of Medicine, Liaquat University of Medical and Health Sciences, Jamshoro, Pakistan; d Department of Medicine, Fatima Jinnah Medical University, Lahore, Pakistan; e Department of Medicine, Allama Iqbal Medical College, Lahore, Pakistan; f Department of Anesthesiology, Mohiuddin Islamic Medical College, Mirpur, Pakistan; g Department of Medicine, Allied Hospital, Faisalabad, Pakistan; h Department of Medicine, Rawalpindi Medical University, Rawalpindi, Pakistan; i Department of Medicine, Khawaja Muhammad Safdar Medical College, Sialkot, Pakistan; j Department of Medicine, King Edward Medical University, Lahore, Pakistan; k Department of Medicine, Sana’a University, Sana’a, Yemen; l Department of Anesthesiology, Geisinger Medical Center, Danville, Pennsylvania

**Keywords:** elderly, endoscopy, propofol, remimazolam

## Abstract

**Background:**

This systematic review and meta-analysis study was undertaken to compare the efficacy and safety of remimazolam versus propofol for sedation in elderly patients (≥60 years old) undergoing gastrointestinal endoscopy.

**Methods:**

The Cochrane Central Register of Controlled Trials, MEDLINE, Embase, and ClinicalTrials.gov were used to perform a thorough literature search from their inception to December 2025. A random-effects meta-analysis was performed using RevMan. The Mantel-Haenszel method was used to pool risk ratios (RRs) along with 95% confidence intervals (95% CIs) for dichotomous outcomes. The inverse variance method was used to pool mean differences (MDs) with 95% CI.

**Results:**

This meta-analysis included 11 RCTs consisting of 2456 participants. Remimazolam significantly reduced the risk of overall adverse events (RR: 0.60, 95% CI: 0.42–0.86), injection site pain (RR: 0.20, 95% CI: 0.12–0.34), hypotension (RR: 0.46, 95% CI: 0.35–0.59), bradycardia (RR: 0.52, 95% CI: 0.34–0.77), need for vasopressors (RR: 0.39, 95% CI: 0.23–0.67), hypoxemia (RR: 0.42, 95% CI: 0.29–0.59), and respiratory depression (RR: 0.42, 95% CI: 0.29–0.62) compared with propofol. There were no statistically significant differences between remimazolam and propofol in sedation success, procedure success, procedure time, onset time, time to full alertness, time to discharge, patient satisfaction, endoscopist satisfaction, postoperative nausea and vomiting, or prolonged sedation.

**Conclusion:**

Our analysis suggests that remimazolam is safer compared to propofol for sedation in endoscopic procedures.

## Introduction

Gastrointestinal (GI) endoscopy is a procedure used to visually examine the GI tract by using a thin, flexible tube equipped with a light and camera (endoscope). It has been considered the gold standard in diagnosing and treating GI diseases in elderly patients (≥60 years old), and it is widely performed worldwide. Endoscopic procedures are invasive and might inflict pain and discomfort on the patient. To relieve patient discomfort and postoperative complications, sedation is increasingly being utilized during this procedure. The use of sedation during endoscopy differs significantly across various countries and regions. A national survey in China showed that there were 10 million gastroscopies performed in 2016. The predicted number will reach 35 million by 2030, according to the growth rate of aging[1]. Older people are at increased risk for adverse events, such as hypoxemia, hypotension, and esophageal reflux, during the examinations[2].

Propofol is a phenolic derivative, has a rapid onset of action, a short half-life, and has become the sedative of choice for short-duration procedures. The use of propofol has been associated with several adverse effects, including respiratory depression, hypotension, and injection pain^[^3,4^]^. Prolonged use of propofol has been associated with propofol-related infusion syndrome (PRIS)[5]. Furthermore, propofol poses a greater risk of hypotension with increasing age beyond 60 years. In older patients (median age ≥60 years), its incidence within the first 10 minutes of anesthesia is significantly higher (*P* < 0.0001) compared to younger individuals[6]. A study found that elderly patients were more susceptible to the adverse effects of propofol, experiencing a significantly higher incidence of adverse events at 31% (*P* = 0.027)[7].

Remimazolam is a short-acting benzodiazepine that acts as a γ-aminobutyric acid (GABA) receptor agonist, which may provide a new direction for sedation. It has a carboxyl ester bond not found in other benzodiazepines, allowing it to be rapidly metabolized into inactive substances by human tissue enzymes[8]. In 2019, the National Medical Products Administration approved its use as a new drug for anesthesia and sedation[9]. Remimazolam has the potential to offer expedited postintervention recovery, setting it apart from propofol. Importantly, owing to its short metabolic half-life, it has demonstrated a superior clinical safety profile and a reduced propensity to induce adverse effects such as respiratory depression, hypotension, and injection pain[10]. Additionally, remimazolam demonstrates significant advantages in alleviating respiratory and circulatory depression due to its pharmacological characteristics[9].

A previous meta-analysis reported that remimazolam was associated with superior safety outcomes but inferior efficacy outcomes compared to propofol[11]. Therefore, the optimal sedative for GI endoscopy in elderly patients remains unclear. Several RCTs have been published in recent years. Consequently, this systematic review and meta-analysis was undertaken to pool all available evidence assessing the efficacy and safety of remimazolam in comparison with propofol in elderly patients undergoing GI endoscopy.

## Materials and methods

Our meta-analysis was reported according to the Preferred Reporting Items for Systematic Reviews and Meta-Analyses (PRISMA) statement[12]. Our protocol is registered with PROSPERO. No artificial intelligence was utilized in this research, as reported using the TITAN guidelines[13].

### Data sources and searches

Cochrane Central Register of Controlled Trials, MEDLINE, Embase, and ClinicalTrials.gov were used to perform a thorough electronic search from their inception to December 2025. Reference lists of included studies and similar systematic reviews were also screened to identify further pertinent studies.

The following terms (“Endoscopy, Gastrointestinal”) AND (“Benzodiazepines”) AND (“Propofol”) were used as either Medical Subject Heading (MeSH) terms or keywords. A detailed search strategy is reported in Supplemental Digital Content Table 1, available at: http://links.lww.com/MS9/B239.

### Eligibility criteria

This meta-analysis included RCTs comparing remimazolam with propofol for sedation before GI endoscopy in elderly patients (>60 years). Parameters for exclusion included any study design other than RCT, such as quasi-randomized trials, observational studies, and studies conducted on animals. No language or date restrictions were applied.

### Study selection and data extraction

We used Rayyan to narrow down and remove duplicates of all the articles yielded by our online search. Two authors independently performed a screening of titles and abstracts to exclude all irrelevant articles. Full-text screening, following our eligibility criteria, was then performed on the remaining studies. Any discrepancies regarding the selection of studies were settled by a third author. Relevant data were extracted into a pre-piloted Excel spreadsheet, which included author name, publication year, country name, sample size, mean age, ASA Grade I/II/III, remimazolam regimen, and propofol regimen.

HIGHLIGHTS

- This meta-analysis included eight RCTs and 2044 patients.
- Remimazolam and propofol were compared for gastrointestinal endoscopy in patients over 60 years old.
- Remimazolam reduced the risk of injection site pain, hypotension, bradycardia, need for vasopressors, and respiratory depression.
- Propofol had a shorter onset time.

### Outcomes

The primary outcome of our study was sedation success and procedure success, while the secondary outcomes were injection site pain, hypotension, procedure time, onset time, time to fully alert, time to discharge, patient satisfaction, endoscopist satisfaction, vasopressor need, postoperative nausea and vomiting, prolonged sedation success, bradycardia, hypoxemia, respiratory depression, and adverse events.

### Risk of bias assessment

The risk of bias in the included studies was evaluated using the revised Cochrane Risk of Bias tool for randomized trials (RoB 2.0)[14]. This tool assesses bias in five domains, which comprise: (1) bias caused by the randomization process; (2) bias due to deviations from intended interventions; (3) bias arising from missing outcome data; (4) bias in the measurement of the outcome; and (5) bias in the selection of the reported result. The risk of bias for each included study was assessed by two investigators, with high, low, or some concerns of bias being reported. Any disagreement was settled by a senior investigator.

### Data synthesis

To conduct the meta-analyses, we used Review Manager (RevMan, Version 5.4.1) software. The Mantel–Haenszel method was used for dichotomous outcomes. Risk ratios (RRs) and corresponding 95% confidence intervals (CIs) were extracted. Mean differences (MDs), along with 95% CIs, were pooled for continuous outcomes using the inverse variance method. A random-effects model was used to carry out the meta-analyses. Pooled estimates were presented as forest plots, and the Higgins *I*² statistic was calculated to evaluate statistical heterogeneity. Leave-one-out sensitivity analyses were undertaken to assess the robustness of our results.

## Results

### Literature search results

**Figure 1.**
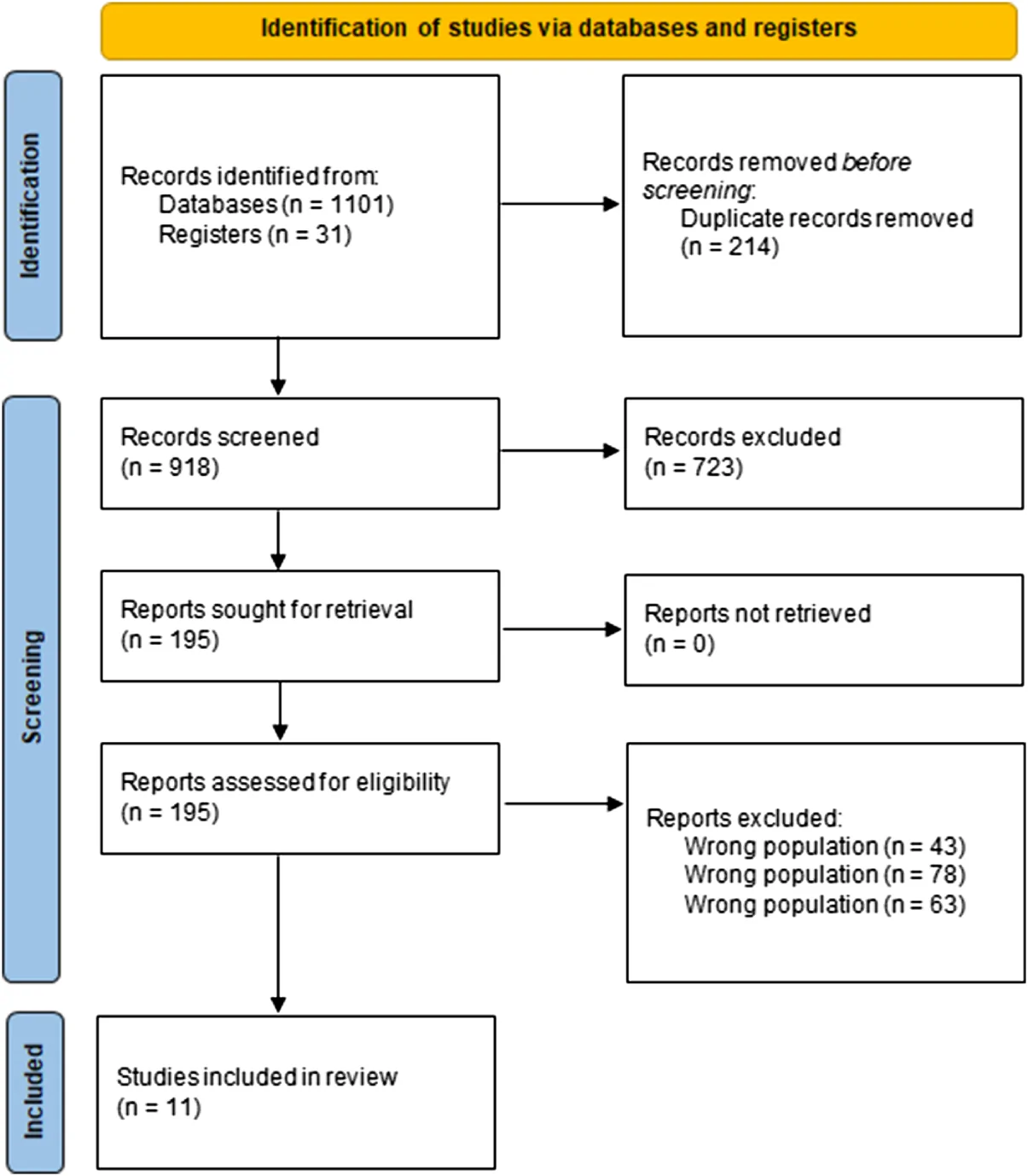
PRISMA Flowchart.

The initial search garnered 1132 articles; 214 duplicates were removed. Title and abstract screening resulted in the exclusion of 723 articles, and 184 out of the remaining 195 studies were excluded after full-text screening. Finally, eleven articles were included in this meta-analysis. The results of the detailed screening process can be observed in the PRISMA flowchart, illustrated in Figure 1.

### Study characteristics

This meta-analysis included eleven RCTs that met the eligibility criteria^[^9,15–24^]^. All RCTs were conducted in China. A total of 2456 patients underwent endoscopy, 1231 patients received remimazolam, and 1195 patients received propofol. The publication years ranged from 2021 to 2024. Outcome definitions varied across trials. Sedation success was assessed using alertness or sedation scales, most frequently MOAA/S or OAA/S. Time to fully alert was defined as the time interval from the final anesthetic dose to a MOAA/S score of 5. Hypotension was predominantly defined as a blood pressure drop of ≥30%. Respiratory depression was defined as a respiratory rate < 8/min and/or SpO₂ < 90%. Injection site pain was defined either through self-report or limb withdrawal. Details of study characteristics are presented in Table 1.

**Table 1:** Characteristics of Included Studies.

Study ID	Country	Sample size	Median age, in years (IQR)	Male (%)	BMI (kg/m^2^)	ASA Grade I/II/III	Remimazolam regimen	Propofol regimen
Chen 2024[17]	China	240 (122 vs 118)	71.9 (5.0) vs 71.7 (5.1)	53.3	22.2 (3.0) vs 22.5 (2.9)	62/178	Remimazolam 0.17 mg/kg + Sufentanil 0.1 µg/kg	Propofol 1.112 mg/kg + Sufentanil 0.1 µg/kg
Chen 2024[19]	China	60 (30 vs 30)	68 (67–72.3) vs 69.5 (66–72)	33	23.3 (3.3) vs 23 (2)	I-III	Remimazolam 6 mg/kg/h until OAA/S of 1 achieved, followed by 5 mg bolus and 1 mg/kg/h continuous infusion	Propofol 18 mg/kg/h until OAA/S of 1 achieved, followed by 6-9 mg/kg/h continuous infusion
Guo 2022[22]	China	82(41 vs 41)	70.4 (66.5–74.3) vs 69.1 (65.1–73.1)	57.3	23 (3.0) vs 23 (3.4)	I-II	Remimazolam 0.15 mg/kg + Alfentanil 5 ug/kg	Propofol 1.5 mg/kg + Alfentanil 5 µg/kg
Hu 2022[20]	China	346 (173 vs 173)	70.1 (71.1–77.38) vs 69.92 (62.35–77.49)	40.75	22.75 (3.15) vs 22.73 (3.23)	20/144/9 vs 26/140/7	Remimazolam 0.2 mg/kg + Sufentanil 0.1 ug/kg	Propofol 1.5 mg/kg + Sufentanil 0.1 µg/kg
Lin 2024[15]	China	228 (114 vs 114)	70 (67–75) vs 70 (68–75)	53.5	22.9 (2.7) vs 22.7 (2.6)	8/79/27 vs 11/73/30	Remimazolam 0.2 mg/kg + Sufentanil 0.05 µg/kg	Propofol 1 mg/kg + Sufentanil 0.05 µg/kg
Liu 2021[24]	China	260 (129 vs 131)	68.87 (2.58) vs 69.12 (2.75)	43	25.35(2.07) vs 24.75(2.16)	I-II	Remimazolam 0.15 mg/kg + Fentanyl 0.5 μg/kg	Etomidate + Propofol 0.1 ml/kg + Fentanyl 0.5 μg/kg
Liu 2023[22]	China	216 (107 vs 109)	67.6 (5.7) vs 67.5 (4.9)	47.2	23.7(3.0) vs 24.0(2.6)	I-II	Remimazolam 0.10 mg/kg + Sufentanil 5ug	Propofol 1.5 mg/kg + Sufentanil 5ug
Lu 2022[23]	China	400 (200 vs 200)	70.6 (4.7) vs 70.1 (4.5)	40.2	22.2 (2.5) vs 22.2 (2.3)	23/37/4	Remimazolam 300 mg/h + Fentanyl 50ųg	Propofol 3.0 g/h + Fentanyl 50ųg
Lu 2024[18]	China	351 (174 vs 177)	69 (67–72)	43.6	24.21 (2.54) vs 24.01 (3.00)	I-II	Remimazolam 0.15 mg/kg + Fetanyl 1 µg/kg	Propofol 1 mg/kg + Fetanyl 1 µg/kg
Tan 2021	China	260 (129-131)	68.87 (2.58) vs 69.12 (2.75)	43	25.35(2.07) vs 24.75(2.16)	I-II	Remimazolam 0.15 mg/kg + Fentanyl Propofol 0.1 ml/kg + Fentanyl 0.1 μg/kg	
Ye 2023[16]	China	129 (64 vs 65)	68 (66–72) vs 68 (66–71)	58.14	22.2 (1.27) vs 22.5 (1.75)	20/44 vs 17/48	Remimazolam 0.2039 mg/kg + remifentanil 0.2 µg/kg	Propofol 1.9733 mg/kg + remifentanil 0.2 µg/kg

### Risk of bias in included studies

The quality assessment of the included studies is presented in Supplemental Digital Content Figure 1, available at: http://links.lww.com/MS9/B238. Overall, eight studies were judged to be at low risk of bias. An inadequate randomization process, deviations from the intended interventions, and measurement of the outcomes led to some concerns of bias in three studies.

### Meta-analysis of primary outcomes

#### Sedation success

**Figure 2.**
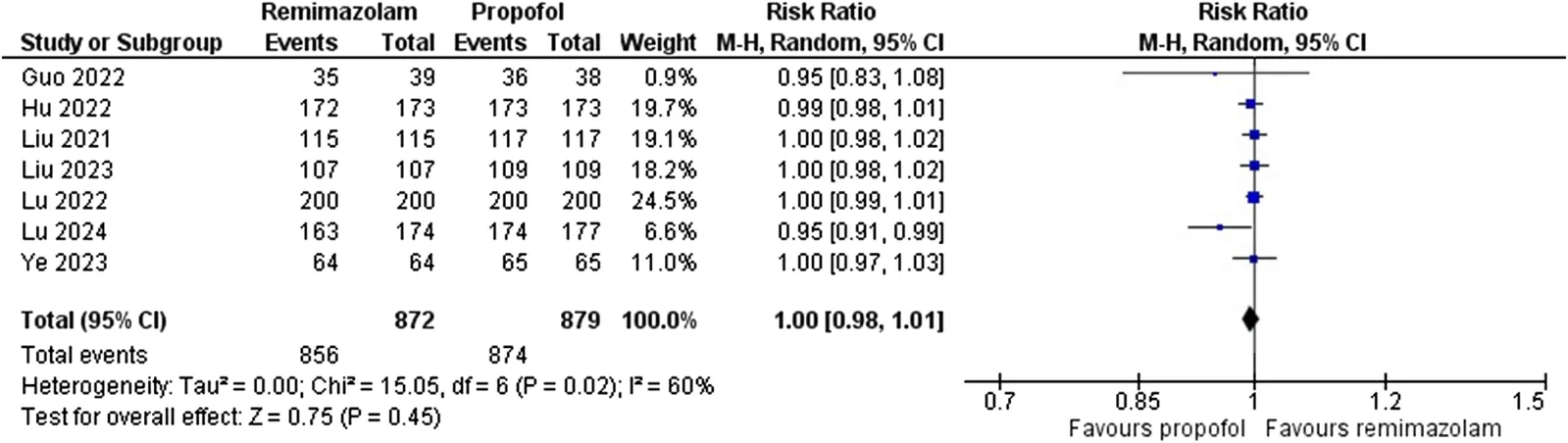
Forest plot of sedation success.

Seven studies reported sedation success. The total number of patients was 1991 (994 remimazolam vs 997 propofol). The results were not statistically significant for this outcome (RR: 0.99, 95% CI: 0.98–1.01, *P* = 0.39, *I*² = 74%) (Fig. 2). Exclusion of Lu *et al* (2024) reduced heterogeneity (*I*² = 45%). Other sensitivity analyses demonstrated comparable findings to the primary analyses.

#### Procedure success

**Figure 3.**
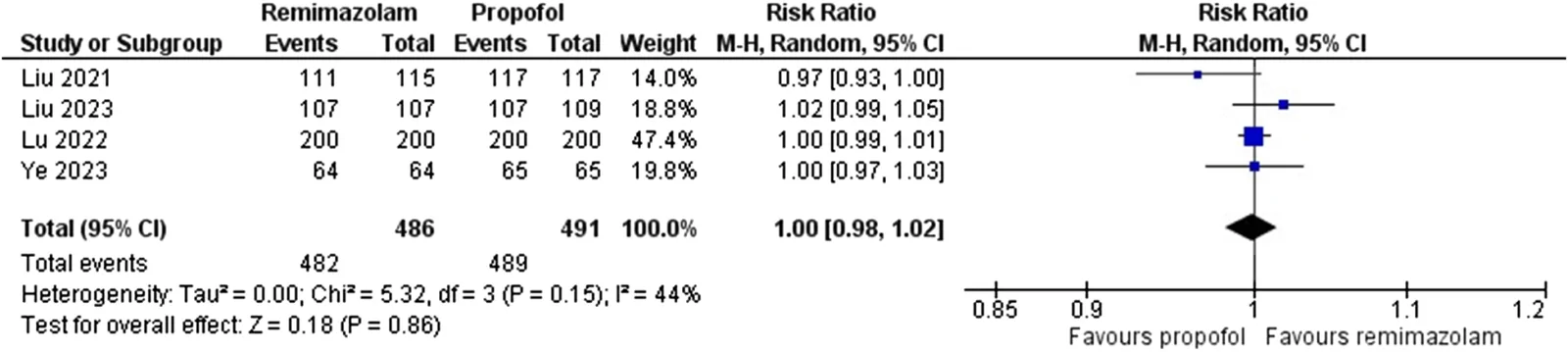
Forest plot of procedure success.

Four studies reported the procedure’s success. The total number of patients was 977 (486 remimazolam vs 491 propofol). Both groups were comparable (RR: 1.00, 95% CI: 0.98–1.02, *P* = 0.86, *I*² = 44%) (Fig. 3). Exclusion of Liu *et al* (2021) reduced heterogeneity (*I*² = 0%). Other sensitivity analyses demonstrated comparable findings to the primary analyses.

### Meta-analysis of secondary outcomes

#### Procedure time

Eight studies reported procedure time. The total number of patients was 1582 (791 remimazolam vs 791 propofol). The results were not significant for this outcome (MD: 0.07, 95% CI: −0.54 to 0.68, *P* = 0.83, *I*² = 64%) (Supplemental Digital Content Figure 2, available at: http://links.lww.com/MS9/B238). Exclusion of Lin *et al* (2024) reduced heterogeneity (*I*² = 5%). Other sensitivity analyses demonstrated comparable findings to the primary analyses.

#### Onset time

Nine studies reported onset time. The total number of patients was 1905 (975 remimazolam vs 930 propofol). The results were not significant for this outcome (MD: 0.39, 95% CI: −0.19 to 0.97, *P* = 0.19, *I*² = 98%) (Supplemental Digital Content Figure 3, available at: http://links.lww.com/MS9/B238). Propofol had a significantly shorter onset time in the leave-one-out sensitivity analysis with the exclusion of Chen *et al* (2024) (MD: 1.03, *P* < 0.00001), Lu *et al* (2022) (MD: 3.42, *P* = 0.04), and Lu *et al* (2024) (MD: 3.42, *P* = 0.04). Other sensitivity analyses demonstrated comparable findings to the primary analyses.

#### Time to be fully alert

Nine studies reported the time to fully alert. The total number of patients was 1794 (923 receiving remimazolam vs 871 receiving propofol). The results were not statistically significant for this outcome (MD: −0.89, 95% CI: −2.03 to 0.25, *P* = 0.13, *I*² = 96%) (Supplemental Digital Content Figure 4, available at: http://links.lww.com/MS9/B238). Sensitivity analyses demonstrated findings comparable to the primary analyses.

#### Time to discharge

Five studies reported the time to discharge. The total number of patients was 1286 (640 remimazolam vs 646 propofol). Both groups were comparable (MD: −0.35, 95% CI: −0.75 to 0.04, *P* = 0.08, *I*² = 36%) (Supplemental Digital Content Figure 5, available at: http://links.lww.com/MS9/B238). Exclusion of Liu *et al* (2021) reduced heterogeneity (*I*² = 0%). Other sensitivity analyses demonstrated comparable findings to the primary analyses.

#### Patient satisfaction

Three studies reported patient satisfaction. The total number of patients was 660 (328 remimazolam vs 332 propofol). The results were not statistically significant for this outcome (MD: 0.00, 95% CI: −0.03 to 0.04, *P* = 0.81, *I*² = 7%) (Supplemental Digital Content Figure 6, available at: http://links.lww.com/MS9/B238). Sensitivity analyses demonstrated comparable findings to the primary analyses.

#### Endoscopist satisfaction

Three studies reported endoscopist satisfaction. The total number of patients was 660 (328 remimazolam vs 332 propofol). Both groups were comparable (MD: 0.00, 95% CI: −0.02 to 0.02, *P* = 0.90, *I*² = 0%) (Supplemental Digital Content Figure 7, available at: http://links.lww.com/MS9/B238). Sensitivity analyses demonstrated comparable findings to the primary analyses.

#### Adverse events

Five studies reported adverse events. The total number of patients was 1336 (667 remimazolam vs 669 propofol). Adverse events were lower with remimazolam (RR: 0.60, 95% CI: 0.42 to 0.86, *P* = 0.005, *I*² = 79%) (Supplemental Digital Content Figure 8, available at: http://links.lww.com/MS9/B238). Sensitivity analyses demonstrated comparable findings to the primary analyses.

#### Injection site pain

Nine studies reported adverse events. The total number of patients was 2063 (1031 remimazolam vs 1032 propofol). The analysis revealed that remimazolam significantly reduced the injection site pain compared to propofol (RR: 0.20, 95% CI: 0.12–0.34, *P* < 0.00001, *I*² = 47%) (Supplemental Digital Content Figure 9, available at: http://links.lww.com/MS9/B238). Sensitivity analyses demonstrated comparable findings to the primary analyses.

#### Hypotension

Ten studies reported hypotension. The total number of patients was 2027 (1030 remimazolam vs 997 propofol). The analysis revealed that remimazolam significantly reduced hypotension compared to propofol (RR: 0.46, 95% CI: 0.35–0.59, *P* < 0.00001, *I*² = 59%) (Supplemental Digital Content Figure 10, available at: http://links.lww.com/MS9/B238). Sensitivity analyses demonstrated comparable findings to the primary analyses.

#### Need of vasopressor

Three studies reported the need for a vasopressor. The total number of patients was 1535 (488 remimazolam vs 490 propofol). The analysis revealed that remimazolam significantly reduced the need for vasopressors compared to propofol (RR: 0.39, 95% CI: 0.23–0.67, *P* = 0.0005, *I*² = 38%) (Supplemental Digital Content Figure 11, available at: http://links.lww.com/MS9/B238). Sensitivity analyses demonstrated comparable findings to the primary analyses.

#### Postoperative nausea and vomiting

Six studies reported hypotension. The total number of patients was 1339 (670 remimazolam vs 669 propofol). The results were not statistically significant for this outcome (RR: 1.12, 95% CI: 0.73 to 1.71, *P* = 0.61, *I*² = 0%) (Supplemental Digital Content Figure 12, available at: http://links.lww.com/MS9/B238). Sensitivity analyses demonstrated comparable findings to the primary analyses.

#### Prolonged sedation

Four studies reported prolonged sedation. The total number of patients was 977 (486 remimazolam vs 491 propofol). Both groups were comparable for this outcome (RR: 0.61, 95% CI: 0.30–1.25, *P* = 0.18, *I*² = 0%) (Supplemental Digital Content Figure 13, available at: http://links.lww.com/MS9/B238). Sensitivity analyses demonstrated comparable findings to the primary analyses.

#### Bradycardia

Seven studies reported bradycardia. The total number of patients was 1611 (803 remimazolam vs 808 propofol). The analysis revealed that remimazolam significantly reduced the risk of bradycardia compared to propofol (RR: 0.52, 95% CI: 0.34–0.77, *P* = 0.001, *I*² = 12%) (Supplemental Digital Content Figure 14, available at: http://links.lww.com/MS9/B238). Sensitivity analyses demonstrated comparable findings to the primary analyses.

#### Hypoxemia

Six studies reported hypoxemia. The total number of patients was 1556 (774 remimazolam vs 782 propofol). The analysis revealed that remimazolam significantly reduced the risk of hypoxemia compared to propofol (RR: 0.42, 95% CI: 0.29–0.59, *P* < 0.00001, *I*² = 0%) (Supplemental Digital Content Figure 15, available at: http://links.lww.com/MS9/B238). Sensitivity analyses demonstrated comparable findings to the primary analyses.

#### Respiratory depression

Five studies reported respiratory depression. The total number of patients was 1303 (650 remimazolam vs 653 propofol). The analysis revealed that remimazolam significantly reduced the risk of respiratory depression compared to propofol (RR: 0.42, 95% CI: 0.29 to 0.62, *P* < 0.00001, *I*² = 2%) (Supplemental Digital Content Figure 16, available at: http://links.lww.com/MS9/B238). Sensitivity analyses demonstrated findings comparable to the primary analyses.

## Discussion

This meta-analysis evaluates the safety and efficacy of remimazolam in comparison to propofol in elderly patients (aged 60 years and above) undergoing gastrointestinal endoscopy and colonoscopy procedures. Our research presents a thorough analysis by aggregating data from eleven RCTs, which produced contentious evidence supporting the superiority of remimazolam compared to propofol. Remimazolam significantly decreases the likelihood of injection site pain, hypoxemia, the requirement for vasopressors, hypotension, bradycardia, and respiratory depression. There were no significant differences between the two groups with respect to postoperative nausea and vomiting, prolonged sedation, endoscopist satisfaction, patient satisfaction, discharge time, time to fully alert, and procedure time. Additionally, sedation success and procedure success were also found to be insignificant between the two groups.

Remimazolam is a water-soluble, ultrashort-acting anesthetic sedative. It undergoes hydrolysis and metabolism through nonspecific tissue esterases. By interacting with the GABA-a receptor, it enhances the opening frequency and permeability of the chloride ion channel in the neuronal membrane. This mechanism facilitates the influx of chloride ions into the cell, driven by a concentration gradient, resulting in an increase in intracellular membrane potential, hyperpolarization, and reduced neuronal excitability. Consequently, this process inhibits neuronal electrical activity, thereby eliciting a sedative effect[25]. Due to its unique methyl propionate side chain mechanism, remimazolam demonstrates a high clearance rate of 1.15 ± 0.12 l/min (mean ± SD), a relatively small steady-state volume of distribution of 35.4 ± 4.2 l, and a brief terminal half-life of 70 ± 10 minutes[26].

Endoscopy patients are more prone to hypotension due to bowel preparation and prolonged fasting. Our results show that hypotension is more common in the propofol group than in the remimazolam group. Propofol is believed to enhance parasympathetic innervation, leading to decreased heart rate (bradycardia) and blood pressure[27], while remimazolam is similar to other benzodiazepine drugs, which may stimulate sympathetic activity and avoid inhibition of cardiopulmonary function[28].

One of the adverse events of propofol sedation is the injection site pain. This pain may decrease the confidence of patients, leading to non-cooperation of the patient during the procedure. There are several factors responsible for the injection site pain caused by propofol. Propofol is an alkylphenol, and all phenols irritate the skin and mucous membranes. The immediate pain can be explained by irritation of the vein endothelium, whereas delayed pain is due to the release of mediators such as kininogen from the kinin cascade[29]. On the other hand, remimazolam reduces injection site pain. This is due to its short metabolic half-life and the blockage of the bradykinin signaling pathway[30]. Our results show injection site pain after propofol administration. This finding is consistent with the previous meta-analysis by Ahmer *et al*, which also shows greater injection site pain after propofol administration[11].

Our analysis showed no significant difference in onset time between remimazolam and propofol. This finding is consistent with a previous meta-analysis that also reported no significant difference in onset time between the two agents[11]. However, our sensitivity analyses demonstrated reduced onset time with propofol following the exclusion of multiple trials. Differences observed across studies may be attributed to variations in patient populations. Our meta-analysis included only elderly patients (aged 60 years and above), whereas earlier meta-analyses included patients from all age groups. Elderly patients are known to be more sensitive to the hypnotic and EEG effects of propofol than younger individuals, and they may require lower sedative doses[11]. Guo *et al* identified that elderly adults may have reduced sedative requirements compared to younger patients[22]. Another meta-analysis demonstrated the superior performance of propofol in terms of onset time in elderly patients[11]. However, onset time remained comparable between remimazolam and propofol in elderly patients in our analysis, which represents the most updated synthesis. Furthermore, our study shows no significant differences between remimazolam and propofol in terms of sedation success and procedure success. However, a previous meta-analysis demonstrates that propofol is slightly superior to remimazolam in terms of sedation success[11]. This can be explained by the variation in dosing regimens across trials. Numerous research studies have shown that different doses of remimazolam induce different levels of sedation, thus affecting the sedation success rate across the trials^[^31–33^]^.

Several limitations should be considered when interpreting the findings of this study. First, the evidence is derived exclusively from randomized controlled trials conducted in elderly patients undergoing gastrointestinal endoscopy, which limits the applicability of the results to younger populations or to other procedural or surgical settings. Second, several included trials were open-label in design, potentially introducing performance and detection bias. Third, all studies were conducted in China, which may restrict the generalizability of the findings due to geographic and practice-pattern differences. Fourth, outcomes were defined and measured differently across studies, which may have influenced pooled estimates. Finally, heterogeneity in study design, including variations in dosing regimens, depth of sedation, and sample sizes, may have contributed to residual confounding across pooled outcomes.

## Conclusion

In conclusion, our meta-analysis of eleven RCTs revealed the comparatively superior safety of remimazolam in terms of adverse events, bradycardia, hypoxemia, respiratory depression, hypotension, and pain on injection. However, no significant differences were found for sedation success, procedure success, procedure time, discharge time, patient satisfaction, endoscopist satisfaction, and nausea and vomiting. Due to the small sample sizes and certain limitations, these findings should be confirmed through future well-designed, large-scale, and double-blinded experimental studies.

## Data Availability

The datasets will be provided by the corresponding author on reasonable request.

## Research registration unique identifying number (UIN)

Prospero: CRD420251043099.
